# Critical action to redress systemic oppression: a person-centered approach

**DOI:** 10.3389/fpsyg.2023.1189598

**Published:** 2023-06-27

**Authors:** Kristin Vierra, Aldo Barrita, Gloria Wong-Padoongpatt, Rachael D. Robnett

**Affiliations:** Department of Psychology, University of Nevada, Las Vegas, Las Vegas, NV, United States

**Keywords:** Black Lives Matter, activism, critical action, performative action, systemic oppression

## Abstract

**Introduction:**

In 2020, public outcry against police brutality prompted many social media users to post black squares and use the hashtag #BlackLivesMatter (BLM). Many of the people who posted these squares were engaging in performative action in the sense that they failed to engage with BLM’s history and core principles. Drawing from a critical action framework, the current research seeks to more deeply understand what drives people to engage in more versus less impactful forms of action to resist systemic oppression.

**Methods:**

We employed a mixed-methods and person-centered methodological approach with the goal of providing nuanced information about factors that distinguish among individuals who engage in different forms of action. Participants were 359 undergraduates who reported that they engaged in some form of action to support BLM.

**Findings:**

Latent profile analysis identified three subgroups (i.e., latent classes) in the larger sample, which we labeled (1) intentional action, (2) intermediate action, and (3) passive action. Participants in each latent class differed from one another in their sociopolitical attitudes, sociodemographic background, and level of action to support BLM. Through the qualitative coding process, the research team unearthed three overarching themes and a range of subthemes that help to explain why the members of each class engaged in different forms of action.

**Discussion:**

We conclude by proposing a flexible intervention that may motivate individuals to engage in critical action to support BLM.

## Introduction

In 2020, public outcry against the social contextual climate surrounding police brutality prompted many social media users to post black squares and use the hashtag #BlackLivesMatter (BLM; Wellman, 2020). Although the posts demonstrated support for BLM, many of the social media users failed to engage with BLM’s history or core principles; instead, they resorted to passive performative activism (Kalina, 2020; Wellman, 2020). The lack of context and depth in the use of the hashtag and black squares minimized the struggle for racial justice and can be perceived as a superficial effort to appear socially aware without truly engaging in meaningful action. The existing scholarship provides limited insight into why some individuals choose to participate in more impactful forms of activism while others opt for superficial or performative action to advocate for people who experience systemic oppression. To this end, the current research focuses on data collected from undergraduates who reported engaging in some form of action to support BLM. Through our mixed-methods and person-centered design, we sought to identify attributes that distinguish among subgroups of participants who engage in more versus less impactful action. In addition to filling a gap in the literature, the research results provide insight into avenues for intervention to motivate individuals to engage in impactful forms of action and become advocates for people who experience systemic oppression.

Previous research has identified various factors that lead individuals to engage in various types of action (Techakesari et al., 2017; Chan and Mak, 2020; Fieck et al., 2020; Goldberg et al., 2020; Moore and Stathi, 2020; Thomas and Newell, 2023). Many of these studies apply either civil courage or critical action as a conceptual framework (Greitemeyer et al., 2007; Broz, 2008; Diemer et al., 2016, 2021; Williams et al., 2023). *Civil courage* is frequently referenced as brave behavior intended to embody or modify ethical norms without concern for the social cost. In addition to advocating for people who experience systemic oppression, civil courage requires knowledge of the specific type of injustice at hand and a willingness to resist social norms associated with this injustice (Williams et al., 2023). On the other hand, *critical action* is defined as individual or collective behaviors taken to create social change and challenge systems of oppression (Diemer et al., 2016). Critical action is an important framework for understanding what motivates some individuals to engage in impactful forms of action compared to potentially performative forms of action. Therefore, the current study focuses on critical action while acknowledging its overlap with the civil courage framework.

The Black Lives Matter global network comprises 40 chapters spanning across the world, all of which share a collective vision for the advancement of Black freedom and a commitment toward liberation efforts (BLMLA, 2023^1^). Moreover, BLM is recognized as the largest social justice movement in US history (The New York Times, 2020^2^). The BLM movement contends that social institutions in the United States, such as the police, devalue Black lives and that the militarization of the police has increased racial oppression. In addition, the BLM organization argues that the police are not the only tool used to oppress and control Black people in America; rather, they see the police as a symptom of a larger issue. They have called for improvements in educational, medical, and political institutions in addition to criminal justice reform (Black Lives Matter, 2023^3^). The present-day civil rights movement relies on action, or public protests, to bring attention to the disproportionate killings of Black people by law enforcement (Garza et al., 2014; Taylor, 2016; Jones-Eversley et al., 2017). Today, the BLM organization continues to critically analyze white supremacy in institutions and motivates its members to continue fighting for Black people’s well-being.

### Distinguishing among different forms of action

Action to support BLM includes a wide array of behaviors. These behaviors include participating in a boycott, protesting for racial justice, volunteering for a political movement in support of BLM, posting on social media, or simply seeking out information about BLM (Hope et al., 2016; Hong and Peoples, 2021; Hendricks et al., 2022). This array of behaviors is reasonable considering collective action is defined as *any* activity intended to improve the circumstances of a group (Wright et al., 1990). Scholars have increasingly argued that action occurs on a spectrum, with some forms of action being more impactful than others (Hope et al., 2016; Techakesari et al., 2017; Chan and Mak, 2020; Fieck et al., 2020; Goldberg et al., 2020; Moore and Stathi, 2020; Hong and Peoples, 2021; Hendricks et al., 2022; Thomas and Newell, 2023).

One less impactful form of action that has been critiqued in recent years is performative action. *Performative action* is described as action that is carried out to build one’s social capital rather than a strong commitment to a social movement (Kalina, 2020; Wellman, 2020). When engaging in performative action, a person would rather let the world know that they are not racist (or sexist, or homophobic) than work to alter the racist institutions that exist in our nation (Kalina, 2020; Wellman, 2020). As mentioned above, in response to police brutality, social media users began uploading black squares and the hashtag #BlackLivesMatter to show support for BLM with little to no context about the organization’s origins. Wellman (2020) found that many social media influencers were deliberately using performative action to gain and keep followers’ trust; generally, their posts did not contribute to meaningful improvements in diversity, equity, or inclusion.

In contrast to performative action, *critical action* is action that has a more meaningful impact. This is because critical action is characterized as a form of civic engagement focused on challenging and dismantling oppressive social systems and is thus not characterized as traditional action (Westheimer and Kahne, 2004; Shaw et al., 2014; Diemer et al., 2021). Critical action is a byproduct of critical reflection (Diemer et al., 2016), which refers to the proficiencies required to critically analyze social systems and support group equity. Once an individual notices oppression within a social system (critical reflection), they are more likely to engage in action to resist and challenge systems of oppression (critical action). Therefore, action that is not accompanied by critical reflection (e.g., posting a black square on social media and returning to life with little or no critical reflection) does not constitute critical action.

To our knowledge, previous research has yet to identify factors that lead individuals to engage in more impactful forms of action (e.g., participating in a protest or march) compared to less impactful forms of action (e.g., posting a black square on social media). Accordingly, the current research uses a mixed-methods design and person-centered approach to identify subgroups of people who engage in impactful versus performative action. In particular, we seek to better understand how the subgroups differ with regard to their backgrounds, traits, life experiences, and reasoning about injustice.

### A person-centered and mixed-methods approach

To address the call for additional research that illuminates why people engage in more versus less impactful forms of action (Diemer et al., 2021), the current research leverages two methodological approaches that are well suited to capturing nuance in the data: a person-centered approach and a mixed-methods approach. *Person-centered* approaches afford insight into meaningful subgroups within a larger sample. For instance, imagine that Lily and Rowan both engage in action to support BLM. Lily posts a black square on social media using the hashtag #BlackLivesMatter. By contrast, Rowan participates in a BLM protest and spends time critically reflecting on systemic oppression. A person-centered approach makes it possible to identify subgroups of people like Lily and Rowan and identify attributes that make these subgroups distinct from one another.

To identify these subgroups in our sample, we utilized latent profile analysis (Hagenaars and McCutcheon, 2002; Muthén and Asparouhov, 2015; Jason and Glenwick, 2016). More specifically, analyses focus on undergraduates who reported engaging in some form of action to support BLM. Through the use of latent profile analysis, we sought to identify subgroups of participants with substantively different profiles on the following correlates: (1) *Critical Consciousness*, which is defined as the ability to critically analyze social and political conditions, approve of societal equity, and engage in action to challenge perceived inequities (Diemer et al., 2017); (2) *Racial colorblindness*, which is defined as the denial or belittlement of race and racism (Neville et al., 2000; Apfelbaum et al., 2012); (3) *Engagement in activism and radicalism*, which is classified as the evaluation of political mobilization and willingness to sacrifice oneself for a group or cause (Moskalenko and McCauley, 2009); (4) *Acknowledgement of white privilege*, which is characterized by awareness of white privilege in America (Mo Bahk and Jandt, 2004); (5) *Everyday discrimination*, which is the belief that one is treated unfairly by individuals and social institutions because of personal characteristics such as race (Williams and Mohammed, 2009; Wong-Padoongpatt et al., 2022); and (6) *Belief in a just world*, which is understood as the belief that good people are rewarded and bad people are punished (Rubin and Peplau, 1975). After deriving the subgroups on the basis of these six correlates, we examined whether the groups differ from one another in terms of the types of action they engaged in to support BLM.

Second, we use a *mixed-methods* approach with the goal of supplementing the quantitative analyses with qualitative data that provides deeper insight into how the participants in each latent class reasoned about engaging in action to support BLM. Incorporating qualitative data to clarify and enrich quantitative patterns is consistent with Creswell’s (2014) sequential mixed-methods design. As highlighted in the most recent American Psychological Association (2017), mixed-methods designs can be helpful in illuminating complex social issues and in capturing individual subjectivities (Grzanka, 2014).

## Materials and methods

The current study is guided by the following objectives: (1) identify subgroups within a sample of undergraduates who engaged in action to support BLM and (2) provide nuanced, in-depth insight into how these subgroups differ from one another. Together, these objectives enable us to shed light on what might contribute to people engaging in more versus less impactful forms of action. We will address the study’s objectives through a blend of quantitative and qualitative data (see Creswell, 2014). First, we will conduct quantitative analyses (namely, latent profile analysis) to identify subgroups (i.e., latent classes) in the sample on the basis of participants’ responses to six scales that assess social attitudes: (1) critical consciousness, (2) racial colorblindness, (3) activism and radicalism, (4) whiteness in America, (5) everyday discrimination, and (6) belief in a just world. We did not have *a priori* hypotheses about the number of classes that would emerge or their attributes, as is typical of person-centered approaches.

After identifying latent classes, our second objective was to examine whether participants in each class differed in meaningful ways. This leads us to Research Question 1: *Do the participants in each latent class differ in terms of their sociodemographic background?* Specifically, we examined whether participants in each latent class varied on the basis of background attributes such as gender, race, and political party. Relatedly, Research Question 2 is as follows: *Do the participants in each latent class engage in different types of action to support Black Lives Matter?* To provide insight into this question, we asked participants about their engagement in various forms of action that ranged from being less impactful (e.g., social media post about BLM) to more impactful (e.g., participating in a BLM protest).

Next, we turned to qualitative data to explore how the participants in each latent class differ from one another when asked to reason about their involvement in action to support BLM. Specifically, Research Question 3 is as follows: *Do the people in different latent classes reason about their action to support BLM in different ways?* In conducting the qualitative analyses, we also explore ethnic-racial differences in how participants reasoned about their engagement in BLM. Specifically, Research Question 4 is as follows: *Compared to the rest of the sample, do Black participants differ in their reasoning for engaging in action to support BLM?*

### Participants

Participants were recruited from a large, public, research-intensive university in the Southwestern U.S. Students at this university have a median family income that is comparable to that of students at other selective public colleges in the region and across the country (The New York Times, 2017^4^). Participants were a subset of a larger sample. Specifically, the current research focuses on 359 undergraduates who reported engaging in some form of action to support BLM. Participants completed an online survey for course credit during the 2020–2022 academic years. All participants were enrolled in introductory psychology, which is a popular general education course. Nearly all participants (91%) were between the ages of 18 and 24. The sample included 243 women (67.7%), 100 men (27.9%), 16 participants (4.5%) who elected not to disclose their gender, and no participants identifying as transgender or gender nonconforming. Participants who elected not to disclose their gender were regretfully not included in the quantitative gender comparisons due to sample size constraints. With regard to political ideology, 198 participants (55.2%) identified as Democrat, 96 participants (26.7%) identified as “Other” or Libertarian, 38 participants (10.6%) identified as Progressive or Socialist, 24 participants (6.7%) identified as Republican, and 3 participants chose not to specify.

With respect to ethnic background, 105 participants (29.2%) identified as Hispanic or Latinx, 80 participants (22.3%) identified as Asian or Pacific Islander, 71 participants (19.8%) identified as white, 59 participants (16.4%) identified as Black or African American, and 44 participants (12.3%) identified as a member of a different ethnic group. Participants who identified as white and as a member of a marginalized group were classified as members of the marginalized group. For example, if a participant identified as white and Black, they were classified as Black. Participants that identified as members of two marginalized ethnic groups (e.g., Black and Latinx) or who identified as members of three or more ethnic groups (e.g., Asian or Pacific Islander, Black, and white) were regretfully not included in the quantitative ethnic comparisons due to sample size constraints. To examine the validity of our self-report measure of ethnic identity, participants were asked what race most people think they are (i.e., street race; see Vargas et al., 2021). Percentages were largely similar apart from white participants: 31 percent selected that people think they are white, whereas our self-report measure placed 20 percent of participants into the white category. A full description of demographic information can be found in Table 1.

**Table 1:** Frequencies and chi-square results for demographic data.

Demographics	Class 1 (%)	Class 2 (%)	Class 3 (%)	*n*	χ^2^	*df*	*p*
Gender				343	22.05		<0.001*
Man	18.2	31.9	50.9				
Woman	81.8	68.1	49.1				
Race				359	14.90	8	0.061
Black/African American	20.9	11.1	17.5				
Asian/Asian American	20.3	27.1	15.8				
Hispanic/Latinx	27.2	33.3	24.6				
White	16.5	19.4	29.8				
Multi-racial	15.2	9.9	12.3				
Political				356	44.74	6	<0.001*
Democrat	58.7	59.7	36.8				
Progressive + Socialist	16.1	8.3	1.8				
Republican	1.9	5.6	22.8				
Libertarian + Other	23.2	26.4	38.6				
Street race				358	14.59	8	0.068
White	29.3	28.5	43.9				
Black	19.1	10.4	15.8				
Asian	20.4	25.7	15.8				
Hispanic/Latinx	26.1	33.3	19.3				
Other	5.15	2.1	5.3				

### Procedure

The university’s introductory psychology subject pool was used to recruit participants for an online survey. All participants provided their consent before beginning the survey. The survey included a short demographic questionnaire, scales assessing participants’ attitudes on social issues, and an open-ended question pertaining to participants’ reasoning for engaging in action to support BLM. As noted, analyses for the current study focus on a subset of participants who reported engaging in some form of action to support BLM. The study described in this manuscript is the only study that utilizes data from this data collection effort. All study materials are available upon reasonable request to the first author. Below, we elaborate on the measures used in the current study.

### Measures

#### Critical consciousness

The critical consciousness scale is a 22-item scale that measures participants’ ability to critically analyze their social and political conditions, approval of societal equity, and engagement in action to challenge perceived inequities (Diemer et al., 2017). For example, participants rated their agreement with statements such as, “Poor people have fewer chances to get ahead.” Participants responded on a scale ranging from 1 (*strongly disagree*) to 6 (*strongly agree*). Higher scores reflect higher critical consciousness. Scores on this measure demonstrated excellent internal reliability (*α* = 0.90).

#### Racial colorblindness

The colorblind racial attitudes scale is a 20-item scale that measures the cognitive aspects of colorblind racial attitudes (Neville et al., 2000). For example, participants responded to items such as, “Everyone who works hard, no matter what race they are, has an equal chance to become rich.” Participants responded on a scale ranging from 1 (*strongly disagree*) to 7 (*strongly agree*). Higher scores reflect higher colorblind racial attitudes. Scores on this measure demonstrated good internal reliability (*α* = 0.81).

#### Activism and radicalism

To evaluate participants’ political mobilization and willingness to sacrifice themselves for a group or cause, we used a 10-item scale created by Moskalenko and McCauley (2009) called the activism and radicalism intention scale. For instance, participants were asked to respond to items such as, “I would travel for an hour to join a public rally, protest, or demonstration in support of my group.” Participants responded on a scale ranging from 1 (*strongly agree*) to 7 (*strongly disagree*). Higher scores indicated that participants were less likely to participate in political mobilization and sacrifice. Scores on this measure demonstrated excellent internal reliability (*α* = 0.91).

#### Whiteness in America

To assess participants’ awareness of white privilege in America, we used a 25-item scale created by Mo Bahk and Jandt (2004) called the whiteness in America scale. An example item is, “White people have privilege in the United States.” Participants responded on a scale ranging from 1 (*strongly disagree*) to 5 (*strongly agree*). Higher scores indicated that a participant was more aware of white privilege in America. Scores on this measure demonstrated adequate internal reliability (*α* = 0.76).

#### Everyday discrimination

To measure participants’ perceived discrimination or belief that they are treated unfairly because of personal characteristics such as race, we used the 9-item everyday discrimination scale (Williams and Mohammed, 2009). Participants responded to items such as, “People act as if they think you are not smart.” Participants responded on a scale ranging from 1 (*almost every day*) to 6 (*never*). Higher scores suggested that participants did not perceive themselves to have experienced discrimination. Scores on this measure demonstrated excellent internal reliability (α = 0.91).

#### Belief in a just world

We measured participants’ personal belief in a just world using the 13-item belief in a just world scale (Rubin and Peplau, 1975). Belief in a just world is generally understood as the belief that good people are rewarded and bad people are punished. Participants responded to items such as, “I believe that, by and large, I deserve what happens to me.” Participants responded on a scale ranging from 1 (*strongly disagree*) to 7 (*strongly agree*). Higher scores indicate greater belief in a just world. Scores on this measure demonstrated good internal reliability (*α* = 0.86).

#### Forms of action

To measure participants’ engagement in action to support BLM, we generated a list of various forms of action through conversation within the research team and in consultation with the literature. This effort led to the identification of 9 distinct types of action: (1) donated money to BLM, (2) posted support on social media for BLM, (3) protested in support of BLM, (4) supported a Black-owned business, (5) volunteered at a nonprofit in support of BLM, (6) education on BLM: e.g., attended a workshop, read a book(s), listened to a podcast(s), (7) signed a petition in support of BLM, (8) voted for politicians or propositions that are pro-BLM, and (9) advocated with friends and family for BLM. Participants were presented with the list and instructed to check all of the action items they had engaged in.

#### Demographics

Participants were asked to share their ethnicity, street race, gender, education level, social-economic status, and political affiliation.

### Qualitative coding

We used an open-ended question to tease out the different forms of reasoning participants used when discussing why they got involved in action. Specifically, participants responded to the following prompt: “Tell us about your most meaningful experience engaging in activities to support Black Lives Matter or similar Anti-racism work. What did you do? Why did you get involved? Did other people you know participate? How did participating impact you?”

We used thematic analysis to code the open-ended data. Thematic analysis is a qualitative method that is used to identify, analyze, and report themes within the data. Braun and Clarke’s (2006) recommendations for thematic analysis guided our analytic approach. First, the lead author thoroughly read through the entire corpus of data and developed a coding manual using a blend of deductive (i.e., theory-driven) and inductive (data-driven) methods. The coding manual included three overarching themes, which each had a number of corresponding coding categories. To test for inter-rater reliability, the lead author and three trained undergraduate research assistants used the coding manual to double-code all 359 responses. The research team met regularly throughout the coding process to establish inter-rater reliability and check for coder drift. Inter-rater reliability, which was indexed by Cohen’s kappa, was good-to-excellent throughout the coding process (*k* = 0.81–0.90). In an effort to supplement our analysis, we tasked research assistants with identifying any types of actions that were not quantitatively captured. However, their analysis did not reveal any additional findings.

### Researcher positionality

Throughout the data analysis and data coding process, the research team sought to be mindful and reflective of the ways in which our positionality may have shaped the questions asked and corresponding analysis. The first author is a doctoral candidate in her 20s who identifies as a White, cisgender woman and whose academic training is in developmental psychology. The second author is a Latinx immigrant and queer doctoral student with an academic training in social, multicultural, and quantitative psychology. The third author is an Asian American woman assistant professor with training in social and multicultural psychology with expertise in microaggressions and addictions. The final author is an associate professor in her 30s who identifies as a White, cisgender woman; her academic background spans developmental, social, and educational psychology. The remaining team members are psychology undergraduate students: (1) A woman in her 20s who identifies as South Asian and bisexual; (2) A woman in her 20s who identifies as Asian/ Filipino and heterosexual; (3) A woman in her 20s who identifies as Mexican and bisexual.

### Analytic approach

The selection of latent profile analysis (LPA) as our analytic approach was motivated by the fact that latent profile analysis is an efficient and powerful technique used to identify “hidden” and meaningfully distinct subgroups in a larger sample. In addition, when compared to other person-centered methodologies like cluster analysis, LPA has clear advantages (Pastor et al., 2007). LPA, in particular, allows researchers to find the optimal number of classes using a model-based approach, reducing the chance of classes being created based on arbitrary or subjective criteria (Pastor et al., 2007). Lastly, LPA allows us to capture Zou and Cheryan’s (2017) suggestion to incorporate dimensionality when examining engagement in activism.

## Results

Creswell (2014) sequential mixed-methods design guided our analytic approach. Within this approach, quantitative findings are prioritized; qualitative findings serve a supportive role by supplementing, clarifying, and contextualizing quantitative patterns. To that purpose, the following analyses are divided into two parts. The results of the latent profile analysis (LPA) are described first, followed by the identification of quantitative correlates of class membership (Research Question 1) and an examination of the different forms of action that characterize each subgroup (Research Question 2). The qualitative data is then used to gain a deeper understanding of the reasoning that might guide participants to engage in different forms of action (Research Questions 3 and 4).

### Latent profile analysis

Descriptive statistics and correlations among study variables are presented in Table 2.

**Table 2:** Descriptive statistics and correlation matrix for continuous variables.

	1	2	3	4	5	6
1. Critical consciousness	--					
2. Racial colorblindness	0.527^***^	--				
3. Activism-radicalism intention	0.199^***^	0.316^***^	--			
4. Everyday discrimination	0.097	0.028	0.125^*^	--		
5. Belief in a just world	0.228^***^	0.117^*^	0.087	0.348^***^	--	
6. Whiteness in America	0.533^***^	0.648^***^	0.226^***^	0.112^*^	0.174^***^	--
Mean	3.50	3.44	3.33	2.58	3.69	3.67
Standard Deviation	0.61	0.94	1.20	1.00	0.96	0.54
Range	1–7	1–7	1–7	1–6	1–7	1–5

^*^p < 0.05; ^***^p < 0.001.

We used LPA to identify individuals who engage in varying levels of action, discover subgroup-level variation between these participants, and examine how the subgroups differ concerning their scores on critical consciousness, racial colorblindness, activism and radicalism, belief in a just world, whiteness in America, and everyday discrimination scales. LPA is a method for categorizing people from a diverse sample into classes (categorical subgroups) that share similar characteristics (Hagenaars and McCutcheon, 2002; Muthén and Asparouhov, 2015). To discover the optimal number of classes, researchers often fit a series of models to which classes are steadily added one by one. Parsimony, conceptual and practical merit, and comparative fit indices are used to determine the best-fitting model (Muthén and Muthén, 2000; Marsh et al., 2009). We relied on a combination of fit indices. Specifically, we considered Akaike’s information criterion (AIC), the Bayesian information criterion (BIC), and the sample-size adjusted Bayesian information criterion (SABIC). Across these fit indices, models with lower values are preferred over models with higher values. We also examined the adjusted likelihood ratio test (LMRT) and the bootstrap likelihood ratio test (BLRT), which indicate whether a model with *k* classes fits significantly better than a model with *k*-1 classes.

Analyses were conducted using M*plus* version 7 (Muthén and Muthén, 2017) using the robust maximum likelihood estimator (MLR). All analyses were run with 3,000 random start values; the best 100 of these starts were retained. We tested latent profile models that encompassed 1, 2, 3, and 4 classes. According to all fit indices, the 2-class model fit significantly better than the 1-class model. Likewise, according to all fit indices the 3-class model fit significantly better than the 2-class model. In contrast, the 4-class model did not fit significantly better than the 3-class model. Therefore, the 3-class model was retained. To further evaluate the robustness of the 3-class solution, we examined the model’s entropy and the latent class probabilities. Both measures have possible values between 0 and 1, with values nearer 1 indicating higher classification quality (Hix-Small et al., 2004; Tein et al., 2013). The 3-class model had an entropy value of 0.73, and the average posterior class membership probabilities were 0.85 (Class 1), 0.90 (Class 2), and 0.86 (Class 3). Taken together, these metrics show that the 3-class solution had sufficient classification quality and accuracy.

**Figure 1:**
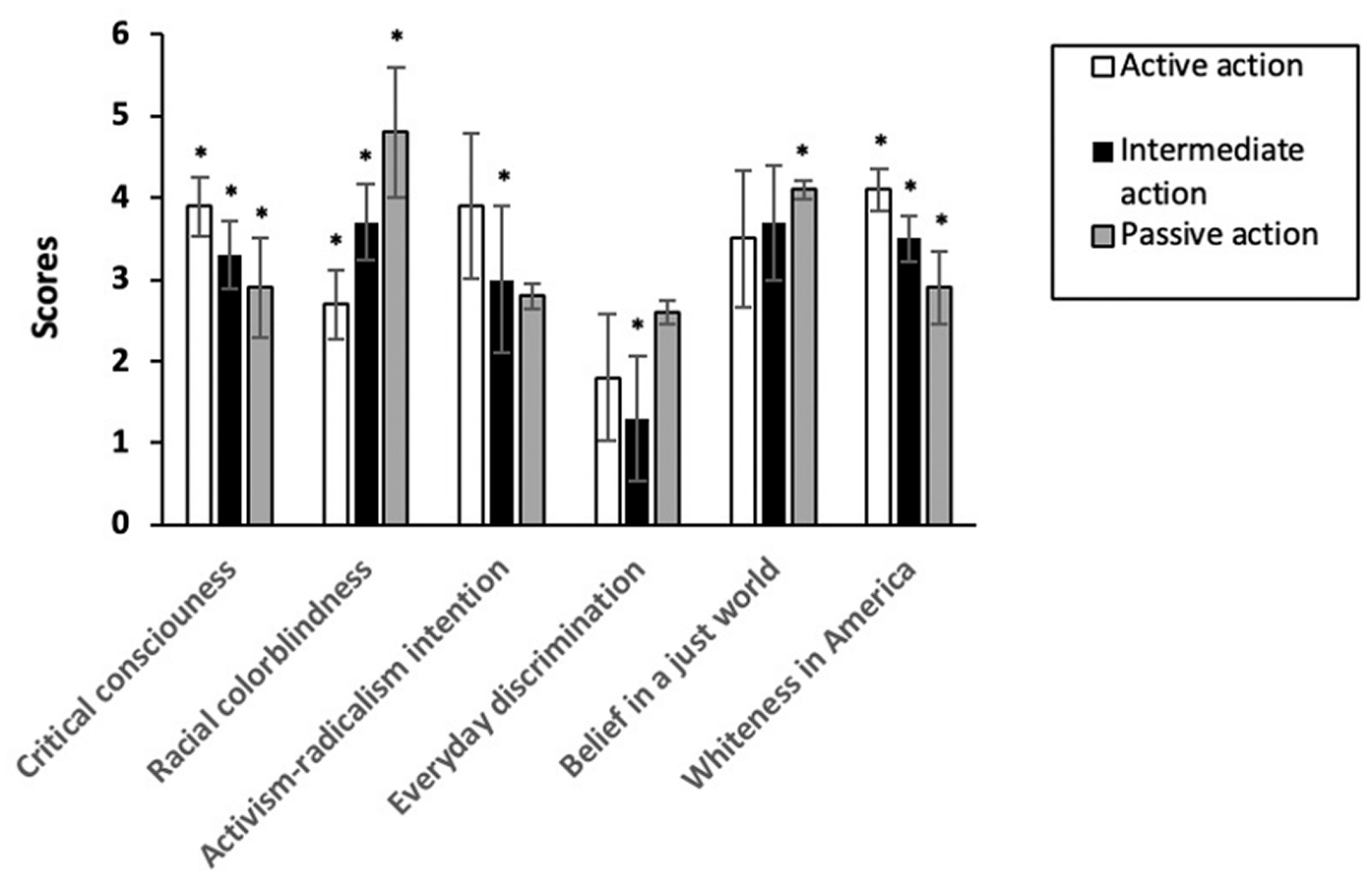
Focal variables scores for different latent classes.

Figure 1 illustrates how the participants in the three latent classes scored on the focal variables: (1) critical consciousness, (2) racial colorblindness, (3) activism and radicalism, (4) whiteness in America, (5) everyday discrimination, and (6) belief in a just world. According to an ANOVA, scores on each focal variable varied significantly by latent class. To further distinguish among the classes, we also used chi-squares to examine (1) whether the members of each class differed in their sociodemographic backgrounds (Research Question 1), (2) whether the members of each class engaged in different forms of action (Research Question 2), and (3) whether members of each class differ in their reasoning about their action to support BLM (Research Questions 3 and 4). Full results of the chi-squares are presented in Tables 1, 3, 4.

**Table 3:** Frequencies and chi-square results for action items supporting BLM.

Action variable	Intentional action (%)	Intermediate action (%)	Passive action (%)	χ^2^	*df*	*p*
Posted on social media	84.80	62.20	52.60	28.25	2	<0.001*
Protested	33.50	10.40	5.30	34.40	2	<0.001*
Supported a Black-owned Business	74.70	56.00	40.40	23.36	2	<0.001*
Volunteered with an anti-racist org	7.00	3.50	3.50	2.30	2	0.325
Educated oneself	64.60	41.70	19.30	38.46	2	<0.001*
Signed a petition	83.50	45.10	26.30	75.99	2	<0.001*
Voted for a politician or policy	57.00	28.50	22.80	33.90	2	<0.001*
Advocated to friends or family	68.40	41.00	29.80	35.06	2	<0.001*

**Table 4:** Frequencies and chi-square results for qualitative themes.

Action variable	Active action (%)	Intermediate action (%)	Passive action (%)	χ^2^	*df*	*p*
Interpersonal	42.90	46.90	10.20	1.83	2	0.401
Structural	56.30	37.50	6.30	1.60	2	0.449
Identity or relationship	19.70	6.90	5.30	14.52	2	<0.001*
Collective efficacy	37.00	55.60	7.40	3.38	2	0.185
Reason for not protesting: Anxiety	41.70	33.30	25.00	0.80	2	0.672
Hierarchy	38.50	53.80	7.70	3.98	2	0.136

#### Class 1

We titled the first latent class *intentional action*. This class was composed of 158 participants (44% of the sample). A Bonferroni post-hoc test (see Figure 1) indicated that compared to participants in the other two classes, participants in the *intentional action* class scored significantly higher on variables such as critical consciousness and whiteness in America. Further, according to the chi-square results (see Table 3), participants in the *intentional action* class were significantly more likely than participants in the other two classes to have engaged in all forms of action except for volunteering for a political movement in support of BLM. In relation to gender and political demographics, 81.8% identified as women, and 58.7% identified as Democrats. Regarding ethnic identity, there was a fairly even distribution of participants from different ethnic groups in this class: 27.2% identified as Hispanic or Latinx, 20.9% identified as Black or African American, 20.3% identified as Asian or Asian American, 16.5% identified as white, and 15.2% identified as more than one marginalized ethnic group.

#### Class 2

We termed the second latent class *intermediate action*. This class was composed of 144 participants (40% of the sample). A Bonferroni post-hoc test (see Figure 1) indicated that participants in the *intermediate action* class scored significantly lower than the intentional action class but significantly higher than the passive action class (Class 3) on variables such as critical consciousness and whiteness in America. According to chi-square results (see Table 3), the *intermediate action* class engaged in all forms of action except for volunteering for a political movement in support of BLM significantly less than the intentional action class but significantly more than the passive action class. In relation to gender and political demographics, 68.1% identified as women, and 81.1% identified as Democrats. Regarding ethnic identity, 33.3% identified as Hispanic or Latinx, 27.1% identified as Asian or Asian American, 19.4% identified as white, 11.1% identified as Black or African American, and 9.9% identified as more than one marginalized ethnic group.

#### Class 3

Lastly, we termed the third-class *passive action*. This class was composed of 57 participants (15% of the sample). A Bonferroni *post hoc* test (see Figure 1) indicated that compared to participants in the other two classes, participants in the *passive action* class scored significantly lower on variables such as critical consciousness and whiteness in America. According to the chi-square results (see Table 3), participants in the *passive action* class were significantly less likely than participants in the other two classes to have engaged in all forms of action except for volunteering for a political movement in support of BLM. In relation to demographics, 49.1% identified as women, and 36.8% identified as Democrats. Regarding ethnic identity, 29.8% identified as white, 24.6% identified as Hispanic or Latinx, 17.5% identified as Black or African American, 15.8% identified as Asian or Asian American, and 12.3% identified as more than one marginalized ethnic group.

### Qualitative analysis

To answer Research Question 3, we turn to our qualitative data to examine whether participants in each latent class differ in their reasoning about their action to support BLM. All themes, coding categories, and sample responses are presented in Table 5. Three overarching themes emerged in the data. The first overarching theme is *reason for engaging in action*. Within this theme, there are four subthemes: (1) interpersonal (*f* = 49), (2) structural (*f* = 16), (3) identity or relationship (*f* = 44), and (4) political efficacy (*f* = 27). The second overarching theme is titled *anxiety as a reason for not protesting* (*f* = 12). The third overarching theme is titled *hierarchy of participation* (*f* = 39). The themes and subthemes were not mutually exclusive, meaning if a participant referenced more than one theme or subtheme, they could be coded into multiple themes or subthemes. A number of qualitative responses were not coded because participants did not provide a rationale for engaging in action to support BLM or simply listed the forms of action they had engaged in with no further detail or context. After coding all responses, we conducted a chi-square analysis to determine whether coding membership differed significantly by class. The results of these analyses are summarized in Table 4 and discussed below as we present each theme.

**Table 5:** Action themes.

Theme	Definition	Example
Reason for Engaging in Action	Participants explained why they engaged in some form of action to support BLM.	“I got involved because I believe in the cause.” (multi-racial woman)
Interpersonal	Participants explained that they engaged in action to resist individual racist interactions, the small number of racist individuals that still exist, or to support the equal treatment of all.	“I got involved because everyone should be treated equal no matter the color of their skin.” (Hispanic or Latinx woman)
Structural	Participants explained that they engaged in action to bring awareness or resist structural systems that create inequalities faced by the Black community. Examples include mentioning police brutality and systematic racism.	“I got involved with protests and the movement because institutional racism is really just sickening and change needed to and still needs to happen.” (multi-racial woman)
Identity or relationship	Participants explained that they engaged in action because of their own ethnic identity or the ethnic identity of someone they are close to. For example, participants may state that they engaged in action because they are a person of color, or they are close to a person of color.	“I got involved because I have experienced discrimination myself, and I have seen even more extreme racism among my African American peers first hand as well.” (Asian or Asian American woman)
Political efficacy	Participants explained that they engaged in action because they felt they could make a positive difference or make a positive impact.	“I felt it was important that I get involved because I wanted to be a part of some kind of change within the community.” (Black man)
Reasoning for not protesting: Anxiety	Participants stated that they did not participate in a BLM protest because they felt unsafe, had anxiety about protesting, were worried about the pandemic, or explained that they do not like to get political.	“I have anxiety in crowds and bigger events, so I was slightly nervous to go out and protest which is why I chose to stay home and post online.” (Hispanic or Latinx woman)
Hierarchy of participation	Participants acknowledged that they could have done more to support BLM. For instance, participants compared themselves to others they believe did more to support BLM, explained that they engaged in less action than they would have hoped to, or used phrases such as: “the most I could do,” “the most I’ve done” or “I would’ve done more but.”	“I know that other people are far more involved than I am in BLM or anti-racism work, but I do try my best to be informed.” (multi-racial woman)

#### Reason for engaging in action

The first overarching theme captures participants’ explanations for why they engaged in some form of action to support BLM. The subthemes that characterize their responses are listed below. A chi-square analysis revealed that participants in the *intentional action class* were significantly more likely that the participants in the other two classes to explain that they engaged in action to support BLM because of their *identity or relationships* (subtheme described below). This means that relative to the rest of the sample, participants in the intentional action class were particularly likely to share that their own ethnic identity or the ethnic identity of someone they know well motivated them to engage in action. All other chi-squares that tested for qualitative variation across the three latent classes were statistically nonsignificant.

##### Interpersonal

Many participants explained that they engaged in action to resist individual racist interactions or the small number of racist individuals that still exist. For example, Darren^5^ (white man) explained that he got involved to combat his family’s unproductive thoughts, stating, “I have a family member who tends to say racist things, which is not ok. I ended up talking to him about things that have been going on with the black community and that he should not just blatantly talk like that about anyone.” In addition, Daniel (Hispanic or Latinx man) explained that he got involved to resist individual racist interactions, stating, “My most meaningful experience in anti-racism work is by protecting minority individuals like myself against rude remarks.” These quotes reflect that some participants are motivated to engage in action to support BLM as a means to resist racism that occurs between individuals without analyzing or resisting social systems that create racial oppression.

The interpersonal theme also captures responses in which participants explained they had engaged in action to support the equal treatment of all. For instance, Ava (white woman) stated, “The reason I got involved was because everyone in our country deserves to be equally treated and not feel like their life is threatened.” Similarly, Mia (Hispanic or Latinx woman) shared, “I got involved because as someone who is in a minority group, I believe everyone should be treated equally and should not be defined because of their race.” These quotes highlight a strong focus on equality (versus equity) and the idea that no matter one’s racial identity, individuals should be given the same resources and opportunities in society.

##### Structural

In contrast to the interpersonal subtheme, participants in the structural subtheme explained that they engaged in action to resist structural systems that create inequities that the Black community faces, such as police brutality. For instance, Maya (Asian or Asian American) voiced that they got involved to protest police brutality, stating, “The reason why I was involved is that the history of police brutality had not stopped.” In addition, Della (Black woman) stated, “I got involved because I had been growing increasingly angry and frustrated with police brutality in the U.S.” Further, some participants referenced unjust structural systems as their reasoning for engaging in action. For instance, Casey (white) expressed motivation to “dismantle harmful stereotypes and reform broken systems.” Moreover, Evelyn (Hispanic or Latinx woman) stated, “I got involved with protests and the movement because institutional racism is really just sickening, and change needed to and still needs to happen.” These quotes indicate that participants hold the ability to construct an analysis of structural oppression. This supports the notion that once participants hold the ability to critically analyze systems of oppression, they are more likely to engage in critical action (Diemer et al., 2016).

##### Identity or relationship

Some participants explained that they engaged in action because of their own ethnic identity or the ethnic identity of someone they know well. For instance, many participants stated that they engaged in action because they are a person of color. Dave (Black man) stated, “I did it because I’m half Black and minorities need to be heard. This made me feel like I might have really impacted change across the world.” Zoey (Black woman) stated, “I got involved because I’m black so seeing black people being murdered in viral videos triggered something in me.” Further, Anna (Hispanic or Latinx woman) stated, “I got involved because not only is the way black people in America are treated completely outrageous, I am also a minority in a position that can help.” Other participants explained that they engaged in action because someone close to them is a person of color. For example, Sarah (Asian or Asian American woman) stated, “I have seen even more extreme racism among my African American peers firsthand as well.”

##### Political efficacy

A number of participants explained that they engaged in action because they felt they could make a positive difference or have an impact on society. For example, Jessie (Asian or Asian American) stated, “I was involved because I wanted the racism to stop. I wanted to make a change.” Jamie (Asian or Asian American man) stated, “I have always wanted to be part of a movement to cause change to those who are oppressed.” These quotes indicate that participants held the motivation and confidence to participate in activities that promote social change.

#### Anxiety as a reason for not protesting

The second overarching theme focuses on a subset of participants who explained that anxiety or related personality attributes motivated them to choose other forms of action over protesting. Participants in this theme frequently mentioned that they did not participate in a BLM protest because they felt unsafe, had anxiety about protesting, were worried about the pandemic, or explained that they do not like to get political. For example, Ariel (multi-racial man) stated,

“I personally do not enjoy getting political and going out in stuff like that. I do know other people that have participated and done protests. However, that’s just not my style of action and how I feel is different.”

Hailey (Latinx woman) stated, “I have anxiety in crowds and bigger events, so I was slightly nervous to go out and protest, which is why I chose to stay home.” These reflections suggest that some participants may require greater political efficacy to hold the motivation and confidence to participate in activities that promote social change. Along a different vein, a handful of participants explained that they did not participate in a BLM protest because of the pandemic. For example, Sara (Hispanic or Latinx woman) explained, “I did not participate in protest for Black Lives Matter because of COVID.”

#### Hierarchy of participation

The third overarching theme focuses on a subset of participants who acknowledged that they could have done more to support BLM. These participants compared themselves to others that they believed had done more to support BLM or explained that they engaged in less action than they would have hoped. For example, Tom (white man) stated, “I felt like I did the bare minimum.” In addition, William (Asian or Asian American man) stated, “Although I shared news and donated, I felt I could not do enough and felt I should’ve done more.” These quotes signify that at least some participants were aware of racial oppression and resistance movements such as BLM, but lacked the direction, motivation, or confidence required to participate more actively in movements such as BLM.

### Ethnic differences in qualitative themes

Lastly, to answer Research Question 4, the qualitative coding team examined whether novel themes emerged when focusing exclusively on Black participants’ qualitative responses. In particular, we sought to examine whether Black participants provided responses that touched on their lived experiences such as describing a sense of exhaustion or social pressure as they attempted to drive the BLM movement forward. However, the qualitative team was unable to identify novel themes that differed from the themes that were present in the broader sample. Below, we discuss how our methodological approach may have made it difficult to tease out meaningful ethnic variation in the qualitative responses.

## Discussion

The purpose of the current study was to identify meaningful subgroups in a sample of undergraduates who engaged in action to support BLM and identify the ways in which these subgroups diffed concerning their backgrounds, traits, life experiences, and reasoning about injustice. We also attempted to better understand what motivates individuals to participate in critical action to become advocates for people who experience systemic oppression. To achieve our objectives, we employed critical action as our conceptual framework (Diemer et al., 2016, 2021) and leveraged a unique blend of person-centered and mixed-methods analyses (see Creswell, 2014).

A latent profile analysis identified three distinct latent classes which were defined by their level of action to support BLM: intentional action (Class 1), (2) intermediate action (Class 2), and passive action (Class 3). Participants in each latent class differed in background traits, life experiences, and reasoning about injustice. In addition to quantitative distinctions among the subgroups, we proposed that people within each latent class may reason differently about social justice and action. Accordingly, we used an open-ended question to tease out these different forms of reasoning. Through the coding process, the research team unearthed three overarching themes and a range of subthemes. The first theme pertains to participants’ *reasons for engaging in action to support BLM*. This theme was characterized by four subthemes, which together capture a range of factors (e.g., desire to resist systemic oppression) that motivated people to engage in action. The second theme pertains to participants’ *reasons for not protesting* wherein participants explained that anxiety or related personality attributes motivated them to favor other forms of action over involvement in BLM protests. The third theme was termed *hierarchy of participation*, wherein participants acknowledge that they could have done more to support BLM.

Below, we elaborate on key findings. Then we draw from our findings to propose a flexible intervention that may encourage individuals to engage in critical action to support BLM. We conclude by speaking on themes of power, discrimination, and privilege.

### Intentional action class

The intentional action class was composed of 158 participants (44% of the sample). There are many unique features of this latent class. Compared to the intermediate and passive action classes, participants in the intentional action class scored higher on constructs that are conceptually associated with social justice (e.g., critical consciousness; Diemer et al., 2017). They also reported higher rates of involvement in *all* forms action to support BLM. This latter pattern is somewhat different than what we anticipated. Specifically, we expected that we might find a subgroup of undergraduates who primarily engaged in critical action while eschewing more performative forms of action. Instead, we identified a subgroup that *takes advantage of all available opportunities* to engage in action—even if some of these forms of action are typically conceptualized as more performative. In the future, it might be fruitful to consider whether behaviors that are sometimes described as performative (e.g., social media posts) are accompanied by more meaningful forms of action.

Another unique attribute of the intentional action class pertains to the qualitative data. Compared to participants in the intermediate and passive action classes, participants in the intentional action class were significantly more likely to explain that they had engaged in action to support BLM due to *their identity or the identity of someone they know well*. Relatedly, although not a statistically significant difference compared to the other two latent classes, there were more Black participants in the intentional action class. Hollander and Einwohner (2004) suggest that Black people may actively engage in acts of resistance against racism as a type of proactive coping. Proactive coping encompasses methods that contest the existence or acceptance of repressive demands made within a racialized system and goes beyond managing one’s own experience with racism. For example, proactive coping seeks to address the detrimental effects of a systemic issue, stress the reality of the injustice, and prioritize group action and motivation over individual action (Aspinwall and Taylor, 1997; Sohl and Moyer, 2009).

### Intermediate action class

We termed the second latent class *intermediate action*. The intermediate action class was composed of 144 participants (40% of the sample). Participants in the intermediate action class scored lower on focal variables compared to the intentional action class but higher than the passive action class. In addition, participants in the intermediate action class engaged in fewer forms of action compared to the intentional action class but more compared to the passive action class. Notably, 33.5% of participants in the intentional action class participated in a BLM protest whereas only 10.4% of participants in the intermediate action class participated in a protest. Although not a statistically significant difference, compared to the other latent classes, there were more Hispanic or Latinx participants in the intermediate action class. This aligns with prior research indicating that trauma-inflicting immigration factors can prompt Hispanic or Latinx families to deter their children from engaging in more noticeable and expressive forms of activism (Suzuki et al., 2022). This may account for the disparity in participating in a protest between the intentional and intermediate action class.

### Passive action class

We termed the third latent class *passive action*. The passive action class was composed of 57 participants (15% of the sample). Compared to the intentional action and intermediate action classes, participants in the *passive action* class scored lower on the focal variables and engaged in fewer forms of action to support BLM. Although not a statistically significant difference, compared to other latent classes, there were more white participants in the passive action class. This is concerning. Freire (2000) argued that liberation of the oppressed requires camaraderie in which the oppressor fights at the side of the oppressed. Taking this into account, future research may benefit from employing a civil courage framework to better understand why white participants fall short when it comes to engaging in critical action to advocate for people who experience systemic oppression.

### Implications for intervention

Taken together, our findings reveal that people engage in various forms of action to support BLM. In addition, people’s level of engagement appears to be associated with their background, life experiences, and reasoning about injustice. Thus, in the forthcoming section, we provide a flexible blueprint of a data-driven intervention that aims to motivate individuals to engage in critical action to support BLM. More specifically, our objective is to use findings from the current research to outline core components of the intervention but allow flexibility in how the intervention will be implemented (Kloos et al., 2012). Because of this flexibility, the intervention can be implemented with participants of various demographic backgrounds and differing readiness to engage in critical action to support BLM.

Our findings demonstrate that scoring low in critical consciousness, activism and radicalism, and whiteness in America act as hurdles to engaging in critical action to support BLM. Further, scoring high in colorblind racial attitudes and belief in a just world also stand as hurdles to engaging in critical action. Therefore, the intervention should start by encouraging *critical thinking*. Critical thinking promotes the skills necessary to recognize systems of oppression, the possible roles one has taken in these systems of oppression, and how one has rationalized the dominant cultural values as accepted truths and norms (Watts et al., 2011). Critical questions will encourage participants to discuss and acknowledge how racial oppression results in an uneven circulation of resources and greatly reduces access to educational opportunities, occupational advancement, and social status for Black individuals. Even more so, these critical questions may increase critical consciousness and whiteness in America and reduce colorblind racial attitudes.

It is also important for intervention facilitators to *break-down the power dynamics* between individual participants. This is essential, considering previous research finds that individuals who hold more social privilege may be more willing to share their perspectives with the group (Howard Jay et al., 2006; Pitt and Packard, 2012; Lee and Mccabe, 2021). This could result in white participants dominating the conversation and not providing a space for participants of color to share their experiences with racial oppression. Further, without a breakdown of power dynamics, this could create pressure for participants of color to primarily serve as a means of facilitating the learning and advancement for white participants (Williams et al., 2020). In addition, it is important that intervention facilitators promote a collective identity amongst intervention members by stressing *respect of all speakers’ opinions*. If participants do not feel safe to open up due to power dynamics or fear of backlash, they may stay silent (Ginwright and Cammarota, 2007). Montero (2009) recommends that interventions encourage active participation from all group members while also showing appreciation for contrasting opinions. Although it is important to appreciate contrasting opinions, Silva (2012) adds that it is essential to respectfully challenge opinions. For example, if a participant shares false information about BLM, it is important that the facilitator remind intervention members of truthful information about BLM. Therefore, we recommend that the intervention facilitator be knowledgeable on the history and goals of BLM.

In the process of hearing diverse viewpoints and opinions from intervention members, individuals will become increasingly *aware of sociopolitical circumstances* (Dillenbourg, 2006; Montero, 2009). This is crucial considering that our findings demonstrated that many participants did not hold a robust understanding of sociopolitical circumstances. Specifically, only a small number of participants mentioned engaging in action to resist structural systems that create inequities. Listening to diverse viewpoints allows participants to piece together varying experiences (unlike their own) and develop a deeper understanding of inequitable sociopolitical circumstances (Dillenbourg, 2006; Montero, 2009). Following participants’ newfound ability to acknowledge and critically analyze racial oppression, participants will, ideally, show (a) greater development of critical consciousness and understanding of white privilege, and (b) reduced endorsement of colorblind racial attitudes and belief in a just world.

After instilling the intervention components delineated above, intervention participants may feel empowered and motivated to take part in critical action (Freire, 2000; Sinacore and Boatwright, 2005). This is especially important considering the *hierarchy of participation* qualitative theme, which revealed a subset of participants who alluded to a desire to engage in more meaningful forms of action. Promoting intentional activism and encouraging participants to engage in authentic discussion may lead to increased engagement in action to support BLM.

### Limitations

As with any study, there are limitations to the current study. Most notably, it is important to revisit Research Question 4 wherein we examined whether Black participants differed from the rest of the sample in their qualitative reasoning for engaging in action to support BLM. This is an intuitive question to investigate considering BLM is arguably more personally meaningful for Black participants. Surprisingly, however, analyses did not reveal substantive ethnic differences in the qualitative data. There are several potential explanations for this unexpected result. First, a small sample of Black participants may be one explanation for the lack of variation. It may be that we simply did not have enough Black participants to capture the nuance in the data required to notice a difference between the Black participants and the rest of the sample. Another explanation may reside in the prompt we used to introduce the open-ended question, which did not directly ask participants to reflect on whether and how their racial-ethnic background played a role in their decision to get involved in action. Relatedly, a member of the qualitative coding team speculated that our use of a short-answer response format might have limited participants’ ability to fully explain their reasoning for engaging in action to support BLM. Specifically, participants may have felt compelled to share their reasoning in just a few sentences. To obtain a more thorough understanding of how ethnic background bears on participants’ reasoning for engaging in action, future research should adopt a methodological approach such as semi-structured interviews that allow for richer, more nuanced responses.

Another limitation pertains to the timing of data collection, which began in 2020. Due to the pandemic, many individuals chose to stay home in 2020. Although a few participants mentioned not protesting due to the pandemic, we did not directly ask about this possibility. Accordingly, we cannot be sure how common it was for participants to avoid protesting due to the pandemic. It is possible that participants will report higher levels of involvement in protests in future research that is not overshadowed by the pandemic. Lastly, due to social desirability or response bias, some participants may have felt pressure to project a favorable image and avoid criticism (Hebert et al., 1997). Therefore, some participants may have responded to survey items falsely or reported engaging in greater amounts of action than was reality.

## Conclusion

The BLM movement has brought to the forefront issues related to power, discrimination, and privilege in contemporary society (Garza et al., 2014; Taylor, 2016; Jones-Eversley et al., 2017). Critical action to support BLM resists the privileged position of those who have historically held power and calls for a redistribution of resources to better support marginalized communities. Our findings demonstrate that in order to become advocates for people who experience systemic oppression and in opposition to those in power, individuals must hold the skills required to critically analyze social systems and support group equity. This study contributes to the literature by providing in-depth information about subgroups within a large sample of undergraduates who engaged in action to support BLM. In addition, our findings provide potential avenues for intervention work. We encourage others to build on our findings through methodological approaches (e.g., interviews) that capture greater nuance in the data with the goal of more deeply understanding how participants’ background shapes their reasoning for engaging in action.

## Data availability statement

The raw data supporting the conclusions of this article will be made available by the authors, without undue reservation.

## Ethics statement

The studies involving human participants were reviewed and approved by The UNLV Office of Research Integrity – Human Subjects. Written informed consent for participation was not required for this study in accordance with the national legislation and the institutional requirements.

## Author contributions

KV helped develop research questions and methodology, performed the quantitative and qualitative analysis, wrote the manuscript, and created the tables and figures. AB helped develop research questions and methodology, and provided feedback on the analysis, interpretation of data, and manuscript. GW-P helped develop research questions and methodology, and provided feedback on the analysis, interpretation of data, and manuscript. RR helped develop research questions and methodology, guided the quantitative and qualitative analysis, and provided feedback on the analysis, interpretation of data, and manuscript. All authors contributed to the article and approved the submitted version.

## Conflict of interest

The authors declare that the research was conducted in the absence of any commercial or financial relationships that could be construed as a potential conflict of interest.

## Publisher’s note

All claims expressed in this article are solely those of the authors and do not necessarily represent those of their affiliated organizations, or those of the publisher, the editors and the reviewers. Any product that may be evaluated in this article, or claim that may be made by its manufacturer, is not guaranteed or endorsed by the publisher.

## Footnotes

^1^ https://www.blmla.org/who-we-are

^2^ https://www.nytimes.com/interactive/2020/07/03/us/george-floyd-protests-crowd-size.html

^3^ https://blacklivesmatter.com/about/

^4^ https://www.nytimes.com/interactive/projects/college-mobility/university-of-nevada-las-vegas

^5^All names are pseudonyms. We present participants’ ethnic background and gender identity alongside their data excerpts when this information is available.

## References

[ref1] American Psychological Association (2017). Multicultural guidelines: an ecological approach to context, identity, and intersectionality. Available at: http://www.apa.org/about/policy/multicultural-guidelines.pdf 10.1037/amp0000382 30762387

[ref2] Apfelbaum E. P. Norton M. I. Sommers S. R. (2012). Racial color blindness: emergence, practice, and implications. Curr. Dir. Psychol. Sci. 21, 205–209. doi: 10.1177/0963721411434980

[ref3] Aspinwall L. G. Taylor S. E. (1997). A stitch in time: self-regulation and proactive coping. Psychol. Bull. 121, 417–436. doi: 10.1037/0033-2909.121.3.417, PMID: 9136643

[ref4] Black Lives Matter (2023). About. Available at: https://blacklivesmatter.com/about/.

[ref5] BLMLA (2023). Who we are. Available at: https://www.blmla.org/who-we-are.

[ref6] Braun V. Clarke V. (2006). Using thematic analysis in psychology. Qual. Res. Psychol. 3, 77–101. doi: 10.1191/1478088706qp063oa

[ref7] Broz S. (2008). Civil courage: good people in an evil time, building and promoting resilience. Afr. Health Sci. 8, S37–S38. PMID: 21448369 PMC3060718

[ref8] Chan R. C. Mak W. W. (2020). Liberating and empowering effects of critical reflection on collective action in LGBT and cisgender heterosexual individuals. Am. J. Community Psychol. 65, 63–77. doi: 10.1002/ajcp.12350, PMID: 31268185

[ref9] Creswell J. W. (2014). Research design: Qualitative, quantitative, and mixed methods approaches. Thousand Oaks, CA: SAGE.

[ref11] Diemer M. A. Pinedo A. Bañales J. Mathews C. J. Frisby M. B. Harris E. M.. (2021). Recentering action in critical consciousness. Child Dev. Perspect. 15, 12–17. doi: 10.1111/cdep.12393

[ref12] Diemer M. A. Rapa L. J. Park C. J. Perry J. C. (2017). Development and validation of the critical consciousness scale. Youth Soc. 49, 461–483. doi: 10.1177/0044118X14538289

[ref13] Diemer M. A. Rapa L. J. Voight A. M. McWhirter E. H. (2016). Critical consciousness: a developmental approach to addressing marginalization and oppression. Child Dev. Perspect. 10, 216–221. doi: 10.1111/cdep.12193

[ref14] Dillenbourg P. (2006). The solo/duo gap. Comput. Hum. Behav. 22, 155–159. doi: 10.1016/j.chb.2005.05.001

[ref15] Fieck M. Miron A. M. Branscombe N. R. Mazurek R. (2020). “We stand up for each other!” an interpretative phenomenological analysis of collective action among US college women. Sex Roles 83, 657–674. doi: 10.1007/s11199-020-01144-y

[ref16] Freire P., (2000). Pedagogy of freedom: Ethics, democracy, and civic courage. Rowman & Littlefield Publishers.

[ref17] Garza A. Tometi O. Cullors P. (2014). A herstory of the# BlackLivesMatter movement. Available at: https://collectiveliberation.org/wp-content/uploads/2015/01/Garza_Herstory_of_the_BlackLivesMatter_Movement.pdf

[ref18] Ginwright S. Cammarota J. (2007). Youth activism in the urban community: learning critical civic praxis within community organizations. Int. J. Qual. Stud. Educ. 20, 693–710. doi: 10.1080/09518390701630833

[ref19] Goldberg S. K. Rothblum E. D. Russell S. T. Meyer I. H. (2020). Exploring the Q in LGBTQ: demographic characteristic and sexuality of queer people in a US representative sample of sexual minorities. Psychol. Sex. Orientat. Gend. Divers. 7, 101–112. doi: 10.1037/sgd0000359, PMID: 34017899 PMC8132578

[ref20] Greitemeyer T. Osswald S. Fischer P. Frey D. (2007). Civil courage: implicit theories, related concepts, and measurement. J. Posit. Psychol. 2, 115–119. doi: 10.1080/17439760701228789

[ref21] Grzanka P. R. (2014). Intersectionality: A foundations and Frontiers Reader. Westview Press, a member of the Perseus Books Group.

[ref22] Hagenaars J. A. McCutcheon A. L. (2002). Applied latent class analysis. Cambridge University Press.

[ref23] Hebert J. R. Ma Y. Clemow L. Ockene I. S. Saperia G. Stanek E. J. III. (1997). Gender differences in social desirability and social approval bias in dietary self-report. Am. J. Epidemiol. 146, 1046–1055. doi: 10.1093/oxfordjournals.aje.a009233, PMID: 9420529

[ref24] Hendricks L. Edwards W. Tietjen-Smith T. Reysen S. (2022). College students’ awareness and familiarity with modern activism: prosocial involvement in black lives matter. J. Hum. Behav. Soc. Environ. 32, 534–547. doi: 10.1080/10911359.2021.1924914

[ref25] Hix-Small H. Duncan T. E. Duncan S. C. Okut H. (2004). A multivariate associative finite growth mixture modeling approach examining adolescent alcohol and marijuana use. J. Psychopathol. Behav. Assess. 26, 255–270. doi: 10.1023/B:JOBA.0000045341.56296.fa

[ref26] Hollander J. A. Einwohner R. L. (2004). “Conceptualizing resistance” in Sociological forum, vol. 19 (Kluwer Academic Publishers-Plenum Publishers), 533–554.

[ref27] Hong P. M. Peoples C. D. (2021). The ties that mobilize us: networks, intergroup contact, and participation in the black lives matter movement. Anal. Soc. Issues Public Policy 21, 541–556. doi: 10.1111/asap.12230

[ref28] Hope E. C. Keels M. Durkee M. I. (2016). Participation in black lives matter and deferred action for childhood arrivals: modern activism among black and Latino college students. J. Divers. High. Educ. 9, 203–215. doi: 10.1037/dhe0000032

[ref29] Howard Jay R. Aimee Z. Yale P. (2006). Students’ race and participation in classroom discussion in introductory sociology: a preliminary investigation. J. Scholar. Teach. Learn. 6, 14–38.

[ref30] Jason L. Glenwick D. (Eds.) (2016). Handbook of methodological approaches to community-based research: Qualitative, quantitative, and mixed methods. New York, NY: Oxford university press.

[ref31] Jones-Eversley S. Adedoyin A. C. Robinson M. A. Moore S. E. (2017). Protesting black inequality: a commentary on the civil rights movement and black lives matter. J. Community Pract. 25, 309–324. doi: 10.1080/10705422.2017.1367343

[ref32] Kalina P. (2020). Performative allyship. Technium Soc. Sci. J. 11, 478–481. doi: 10.47577/tssj.v11i1.1518

[ref33] Kloos B. Hill J. Thomas E. Wandersman A. Elias M. J. Dalton J. H. (2012). Community psychology. Belmont, CA: Cengage Learning.

[ref34] Lee J. J. Mccabe J. M. (2021). Who speaks and who listens: revisiting the chilly climate in college classrooms. Gend. Soc. 35, 32–60. doi: 10.1177/0891243220977141

[ref35] Marsh H. W. Muthén B. Asparouhov T. Lüdtke O. Robitzsch A. Morin A. J.. (2009). Exploratory structural equation modeling, integrating CFA and EFA: application to students’ evaluations of university teaching. Struct. Equ. Model. Multidiscip. J. 16, 439–476. doi: 10.1080/10705510903008220

[ref36] Mo Bahk C. Jandt F. E. (2004). Being white in America: development of a scale. Howard J. Commun. 15, 57–68. doi: 10.1080/10646170490275332

[ref37] Montero M. (2009). “Methods for liberation: critical consciousness in action” in Psychology of liberation. eds. Sonn C. Montero M. (New York, NY: Springer), 73–91.

[ref38] Moore A. Stathi S. (2020). The impact of feminist stereotypes and sexual identity on feminist self-identification and collective action. J. Soc. Psychol. 160, 267–281. doi: 10.1080/00224545.2019.1644280, PMID: 31322058

[ref39] Moskalenko S. McCauley C. (2009). Measuring political mobilization: the distinction between activism and radicalism. Terror. Polit. Viol. 21, 239–260. doi: 10.1080/09546550902765508

[ref40] Muthén B. Asparouhov T. (2015). Causal effects in mediation modeling: an introduction with applications to latent variables. Struct. Equ. Model. Multidiscip. J. 22, 12–23. doi: 10.1080/10705511.2014.935843

[ref41] Muthén B. Muthén L. K. (2000). Integrating person-centered and variable-centered analyses: growth mixture modeling with latent trajectory classes. Alcohol. Clin. Exp. Res. 24, 882–891. doi: 10.1111/j.1530-0277.2000.tb02070.x, PMID: 10888079

[ref42] Muthén B. Muthén L. (2017). “Mplus” in Handbook of item response theory (Chapman and Hall/CRC), 507–518.

[ref43] Neville H. A. Lilly R. L. Duran G. Lee R. M. Browne L. (2000). Construction and initial validation of the color-blind racial attitudes scale (CoBRAS). J. Couns. Psychol. 47, 59–70. doi: 10.1037/0022-0167.47.1.59

[ref44] Pastor D. A. Barron K. E. Miller B. J. Davis S. L. (2007). A latent profile analysis of college students’ achievement goal orientation. Contemp. Educ. Psychol. 32, 8–47. doi: 10.1016/j.cedpsych.2006.10.003

[ref45] Pitt R. N. Packard J. (2012). Activating diversity: the impact of student race on contributions to course discussions. Sociol. Q. 53, 295–320. doi: 10.1111/j.1533-8525.2012.01235.x, PMID: 22616119

[ref46] Rubin Z. Peplau L. A. (1975). Who believes in a just world? J. Soc. Issues 31, 65–89. doi: 10.1111/j.1540-4560.1975.tb00997.x

[ref47] Shaw A. Brady B. McGrath B. Brennan M. A. Dolan P. (2014). Understanding youth civic engagement: debates, discourses, and lessons from practice. Community Dev. 45, 300–316. doi: 10.1080/15575330.2014.931447

[ref48] Silva J. M. (2012). Critical classrooms: using artists’ lives to teach young students social groups, power, and privilege. Urban Educ. 47, 776–800. doi: 10.1177/0042085912441187

[ref49] Sinacore A. L. Boatwright K. J. (2005). “The feminist classroom: feminist strategies and student responses” in Teaching and social justice: integrating multicultural and feminist theories in the classroom. eds. Enns C. Z. Sinacore A. L. (Washington, DC: American Psychological Association), 109–124.

[ref50] Sohl S. J. Moyer A. (2009). Refining the conceptualization of a future-oriented self-regulatory behavior: proactive coping. Personal. Individ. Differ. 47, 139–144. doi: 10.1016/j.paid.2009.02.013, PMID: 19578529 PMC2705166

[ref51] Suzuki S. Quiles T. B. Maker Castro E. (2022). Critical action among Asian and Hispanic/Latinx youth: identifying a multidimensional measure and exploring within-group differences. J. Community Appl. Soc. Psychol. 33, 406–424. doi: 10.1002/casp.2660 37089189 PMC10121194

[ref52] Taylor K. Y. (2016). From# BlackLivesMatter to black liberation. Haymarket Books.

[ref53] Techakesari P. Droogendyk L. Wright S. C. Louis W. R. Barlow F. K. (2017). Supportive contact and LGBT collective action: the moderating role of membership in specific groups. Peace Conflict 23, 307–316. doi: 10.1037/pac0000240

[ref54] Tein J. Y. Coxe S. Cham H. (2013). Statistical power to detect the correct number of classes in latent profile analysis. Struct. Equ. Model. Multidiscip. J. 20, 640–657. doi: 10.1080/10705511.2013.824781, PMID: 24489457 PMC3904803

[ref55] The New York Times (2017). Economic diversity and student outcomes at the University of Nevada, Las Vegas. [online] Available at: https://www.nytimes.com/interactive/projects/college-mobility/university-of-nevada-las-vegas

[ref56] The New York Times (2020). Black lives matter may be the largest movement in U.S. history. Available at: https://www.nytimes.com/interactive/2020/07/03/us/george-floyd-protests-crowd-size.html.

[ref57] Thomas J. J. Newell E. E. (2023). What motivates action for gender quality among emerging adult women? The importance of critical reflection, efficacy, and feminist identity. J. Genet. Psychol. 184, 42–54. doi: 10.1080/00221325.2022.2115337, PMID: 36002339

[ref59] Vargas E. D. Juarez M. Stone L. C. Lopez N. (2021). Critical ‘street race’ praxis: advancing the measurement of racial discrimination among diverse Latinx communities in the US. Crit. Public Health 31, 381–391. doi: 10.1080/09581596.2019.1695040

[ref60] Watts R. J. Diemer M. A. Voight A. M. (2011). Critical consciousness: current status and future directions. New Dir. Child Adolesc. Dev. 2011, 43–57. doi: 10.1002/cd.310, PMID: 22147600

[ref61] Wellman M. L. (2020). Black squares for black lives? Performative allyship as credibility maintenance for social media influencers on Instagram. Soc Media+ Soc 8:205630512210804. doi: 10.1177/20563051221080473

[ref62] Westheimer J. Kahne J. (2004). What kind of citizen? The politics of educating for democracy. Am. Educ. Res. J. 41, 237–269. doi: 10.3102/00028312041002237

[ref63] Williams M. T. Faber S. Nepton A. Ching T. H. (2023). Racial justice allyship requires civil courage: a behavioral prescription for moral growth and change. Am. Psychol. 78, 1–19. doi: 10.1037/amp0000940, PMID: 35143235

[ref64] Williams M. T. Kanter J. W. Peña A. Ching T. H. Oshin L. (2020). Reducing microaggressions and promoting interracial connection: the racial harmony workshop. J. Contextual Behav. Sci. 16, 153–161. doi: 10.1016/j.jcbs.2020.04.008

[ref65] Williams D. R. Mohammed S. A. (2009). Discrimination and racial disparities in health: evidence and needed research. J. Behav. Med. 32, 20–47. doi: 10.1007/s10865-008-9185-0, PMID: 19030981 PMC2821669

[ref66] Wong-Padoongpatt G. Barrita A. King A. (2022). Everyday racism increase for Asians in the U.S. during the COVID-19 pandemic. Asian Am. J. Psychol. 13, 318–327. doi: 10.1037/aap0000295

[ref67] Wright S. C. Taylor D. M. Moghaddam F. M. (1990). Responding to membership in a disadvantaged group: from acceptance to collective protest. J. Pers. Soc. Psychol. 58, 994–1003. doi: 10.1037/0022-3514.58.6.994

[ref68] Zou L. X. Cheryan S. (2017). Two axes of subordination: a new model of racial position. J. Pers. Soc. Psychol. 112, 696–717. doi: 10.1037/pspa0000080, PMID: 28240941

